# Supplementary material for: Free energies of membrane stalk formation from a lipidomics perspective
Source: Nat Commun. 2021 Nov 15;12:6594. doi: 10.1038/s41467-021-26924-2 (PMC8593120; doi:10.1038/s41467-021-26924-2)
Supplement: Supplementary file 1 — Supplementary Information [file 41467_2021_26924_MOESM1_ESM.pdf]

Supplementary information for: Free energies of membrane stalk formation from a lipidomics perspective

Chetan S. Poojari[1], Katharina C. Scherer[1], and Jochen S. Hub[1*]

[1] *Theoretical Physics and Center for Biophysics, Saarland University, Saarbrücken, Germany*

E-mail: jochen.hub@uni-saarland.de

Phone: +49 (0)681 302-2740. Fax: +49 (0)681 302-2748

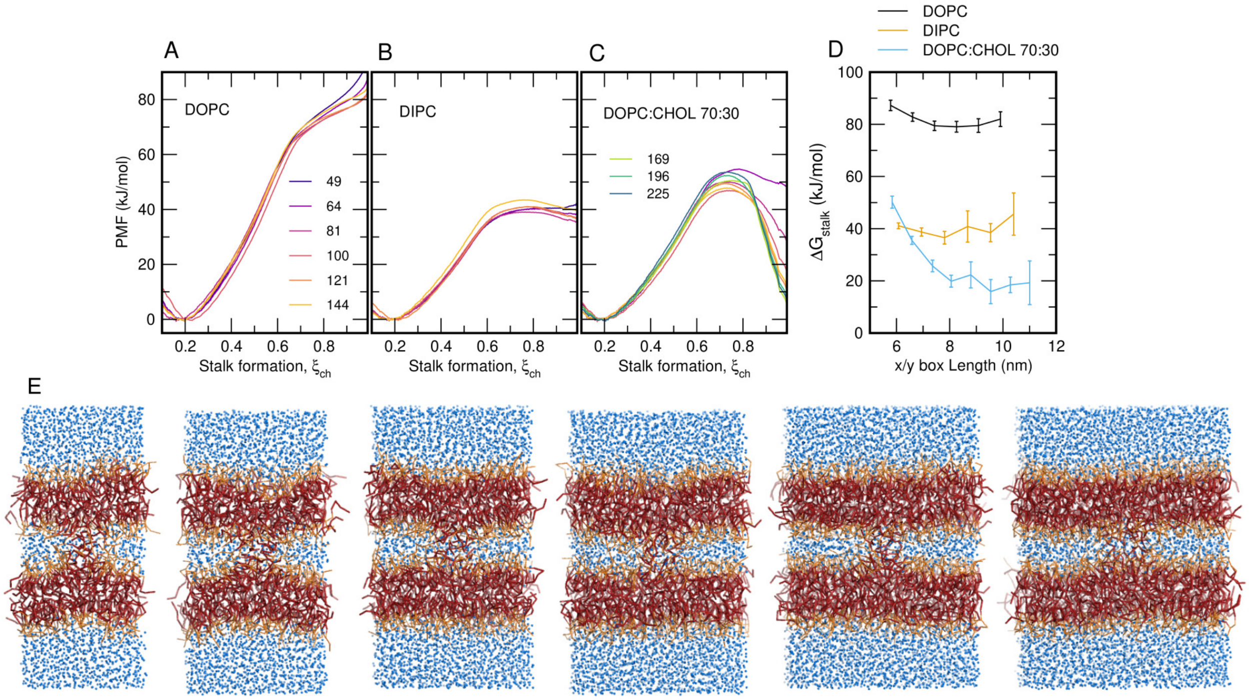

Supplementary Figure 1: Influence of simulation system size on PMFs of stalk formation for (A) DOPC, (B) DIPC, and (C) a 70:30 mixture of DOPC and cholesterol. PMFs were computed using 49 through 225 lipids per monolayer (196 through 900 lipids in total), see color codes. The proximal compartment was hydrated with 10 water beads per nm^2. (D) Free energy of the stalk ΔG_{stalk} versus the box length, revealing that cholesterol-containing simulations require slightly larger systems to avoid artifacts from periodic boundaries. Error bars denote two standard errors. ΔG_{stalk} values were taken from the PMFs at $\xi_{\text{ch}} = 0.95$. (E) Simulation frames of DIPC simulations with a fully formed stalk composed of (from left to right) 49, 64, 81, 100, 121, or 144 lipids per monolayer. Frames were taken from the final snapshots of umbrella sampling simulations restrained to the state of the open stalk ($\xi_{\text{ch}} = 1$). Error bars computed by bootstrapping (see Supplementary Methods) denote 2 SE. Source data are provided as a Source Data file.

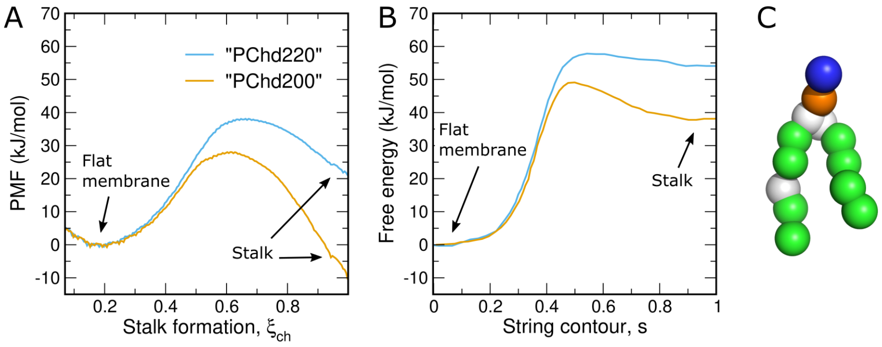

Supplementary Figure 2: Comparison of (A) the PMF along the chain coordinate ξ_{ch} with (B) the minimum free energy path (MFEP) for stalk formation presented by Smirnova *et al.*,[1] computed along an order parameter given the three-dimensional (3D) hydrophobic membrane density and optimized with the string method. Curves in (B) were taken from Ref. 1. PMFs in panel (A) and MFEPs in (B) were computed with the same simulation systems and topologies, kindly provided by the authors of Ref. 1. (C) For the profiles in panels (A) and (B), an older MARTINI POPC model with a 5-bead oleoyl tail was used for both simulations, longer than the four-bead oleoyl model used for all other simulations of this study. The systems contained 128 POPC lipids per bilayer and either 200 (PChd200) or 220 (PChd220) water beads in the proximal compartment, corresponding to 1.56 or 1.72 water beads per lipid. Evidently, the PMFs along ξ_{ch} suggest lower free energies for the stalk as compared to the MFEPs along the density-based order parameter used in Ref. 1. The difference of $\sim 30\,\mathrm{kJ/mol}$ may be rationalized by the different definitions of the end states. In our method, the stalk end state ($\xi_{ch} \approx 1$) is defined by the presence of a hydrophobic connection between the two membranes, but all possible shapes, radii, and lateral positions of the connection are included in the state with $\xi_{ch} \approx 1$. In Ref. 1, the stalk state is defined with a specific 3D density of the hydrophobic beads, which may allow fewer conformational states than the stalk definition adopted by us.[2] Source data are provided as a Source Data file.

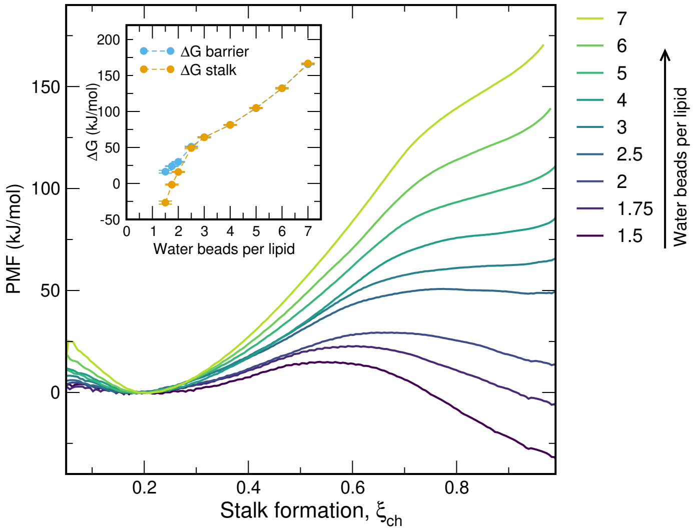

Supplementary Figure 3: PMFs of stalk formation for membranes of pure POPC, computed with the beta-3.2 release of MARTINI 3.0. PMFs were computed with increasing amount of water in the proximal water compartment, defined by the number of water beads per lipid (see legend). Inset: Free energy of the stalk ΔG_{stalk} and of the stalk nucleation barrier ($\Delta G_{\text{barrier}}$, if present) versus water beads per lipid in the proximal compartment, as taken from the PMFs. Error bars computed by bootstrapping denote 1 SE. Source data are provided as a Source Data file.

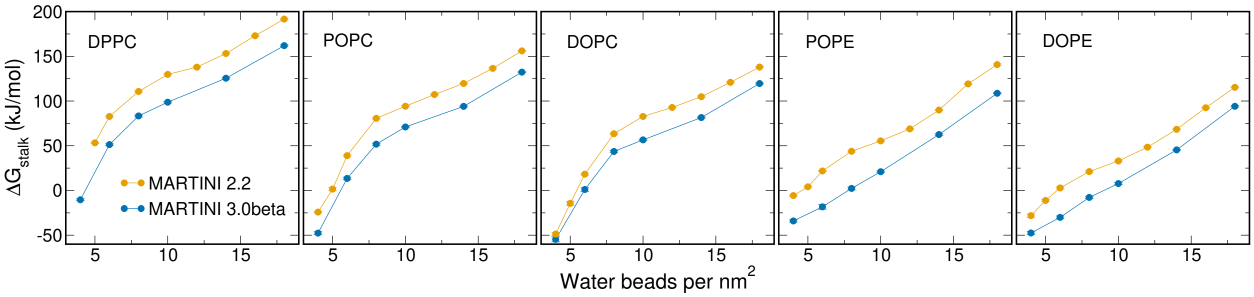

Supplementary Figure 4: Free energies of stalk formation ΔG_{stalk} for five lipid types (see labels) for various degrees of hydration in the proximal compartment. PMFs were computed with the Martini 2.2 model (yellow) or with the beta 3.2 release of Martini 3. The trends of ΔG_{stalk} with hydration, tail unsaturation, and head group favorable agree among the two models. However, the beta release of Martini 3 yields systematically lower ΔG_{stalk}, implying more fusogenic membranes. Error bars computed by bootstrapping denote 1 SE. Source data are provided as a Source Data file.

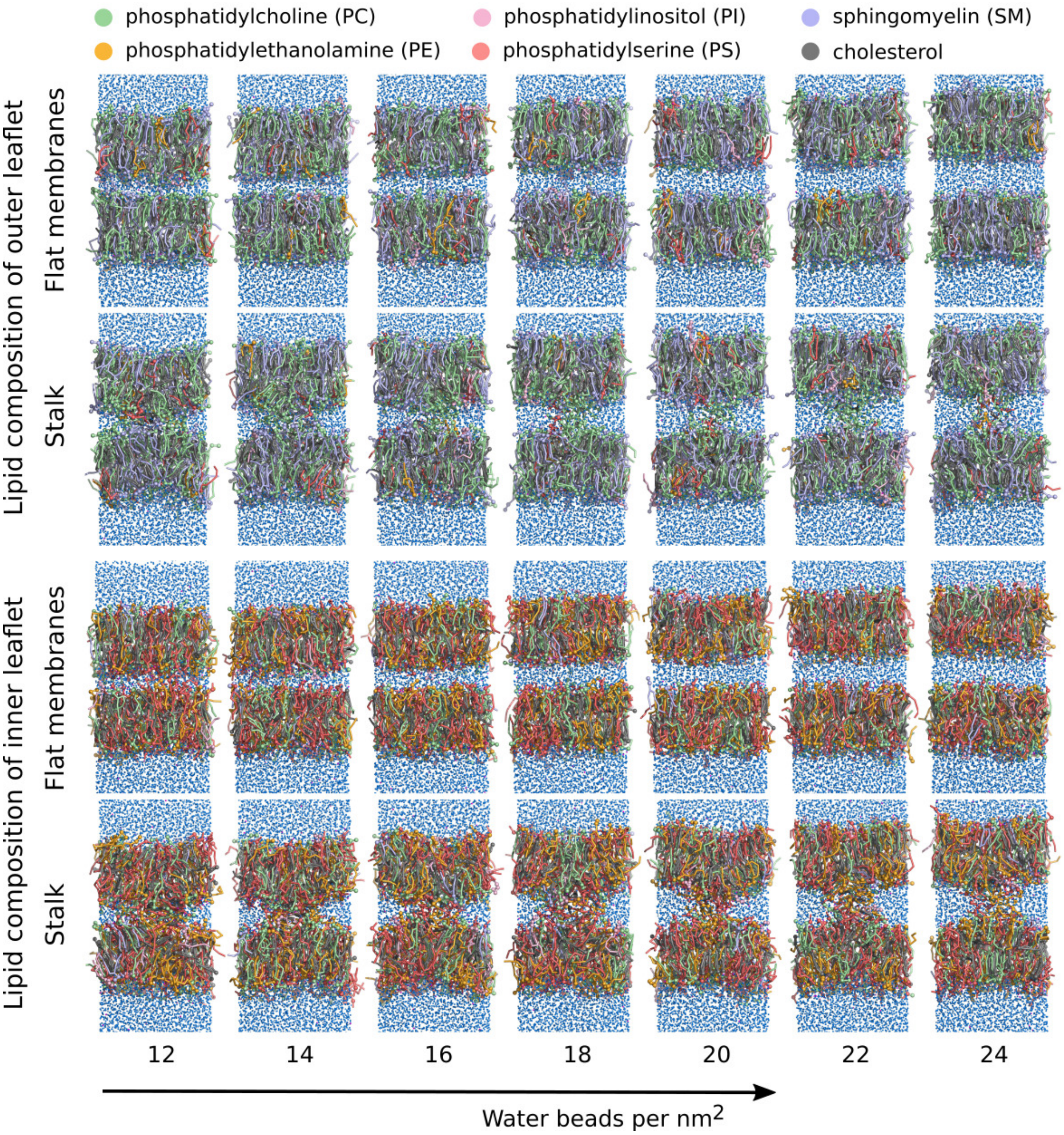

Supplementary Figure 5: Simulations of stalk formation between membranes with the plasma membrane lipid composition. Upper two rows: lipid composition of the outer leaflet; lower two rows: lipid composition of the inner leaflet. From left to right: simulations systems with increasing hydration in the proximal water compartment, between 12 and 24 water beads per nm^2. Lipids are shown as sticks, water and Na$^+$ beads as blue and magenta spheres, respectively. The color of the lipids indicates the lipid type, see legend. Simulation frames were taken from the final snapshots of umbrella sampling simulations restrained to the state of two flat membranes ($\xi_{\mathrm{ch}} = 0.2$) or to the state of the open stalk ($\xi_{\mathrm{ch}} = 1$), respectively. Source data are provided as a Source Data file.

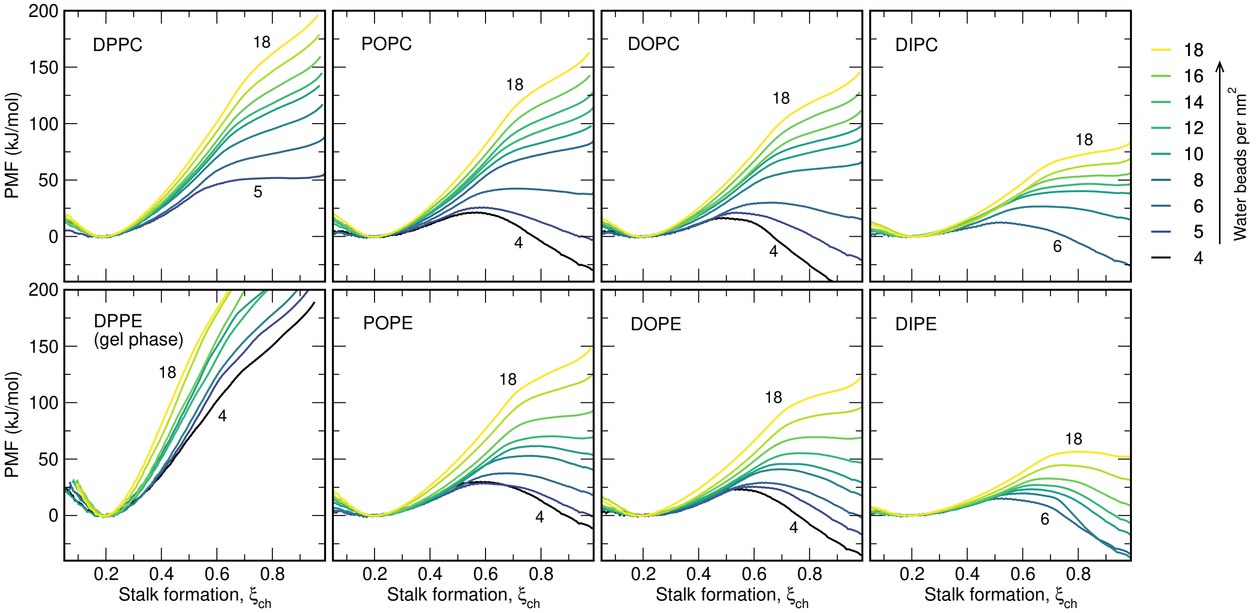

Supplementary Figure 6: PMFs of stalk formation for PC lipids (upper row) and PE lipids (lower row) with increasing tail unsaturation (from left to right), computed with the MARTINI 2.2 model. ΔG_{stalk} for PE membranes is mostly lower as compared to PC membranes. Exceptions are the ΔG_{stalk} values at very low hydration, such as DOPC versus DOPE at 4–5 water beads/nm^2. Another exception is given by DPPE membranes, which formed a gel phase in the simulations, leading to greatly increased ΔG_{stalk} values and highly unstable stalks. Source data are provided as a Source Data file.

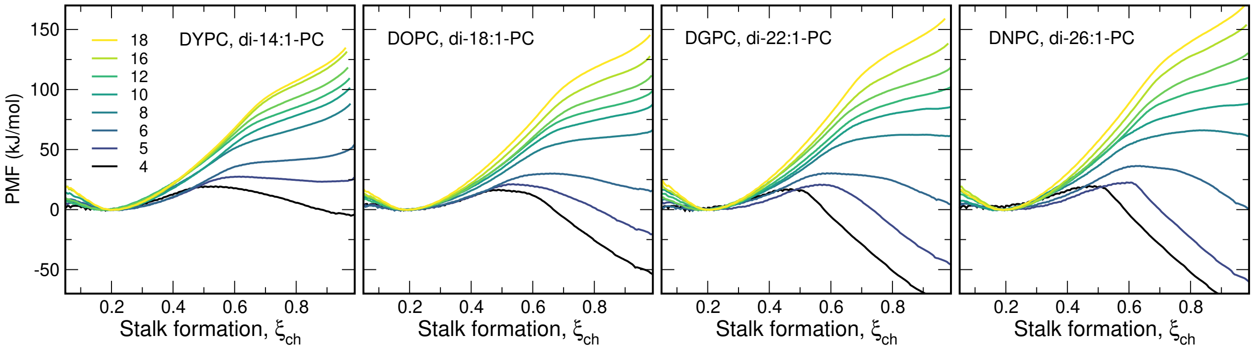

Supplementary Figure 7: On the effect of tail length on the free energies of stalk formation: PMFs of stalk formation with membranes of (from left to right) DYPC, DOPC, DGPC, and DNPC. These MARTINI lipid types correspond approximately to atomistic lipids di-14:1-PC, di-18:1-PC, di-22:1-PC, and di-26:1-PC, respectively. The color code (from black to yellow) indicates increased hydration from 4–18 water beads per nm^2 in the proximal water compartment. Simulation frames of the open stalk for system with 6 water beads/nm^2 are shown in Fig. 9. Source data are provided as a Source Data file.

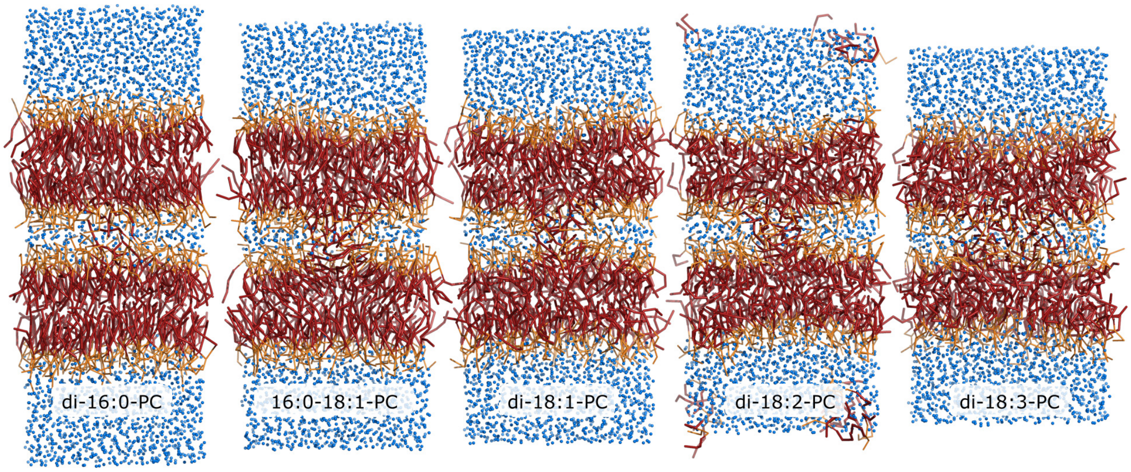

Supplementary Figure 8: Typical simulation frame with an open stalk across membranes with increasing tail unsaturation. From left to right: of DPPC, POPC, DOPC, DLiPC, and DFPC. These MARTINI lipid types contain 4 beads and correspond approximately to atomistic lipids di-16:0-PC, di-16:0-18:1-PC, di-18:1-PC, di-18:2-PC, di-18:3-PC respectively. The proximal water compartment contains 6 water beads per nm^2. Frames were taken from the final snapshot of the last umbrella sampling window restrained to $\xi_{ch} = 1$. Lipid tails are shown as dark red sticks, head groups and glycerol region as orange sticks, and water beads as blue spheres. Source data are provided as a Source Data file.

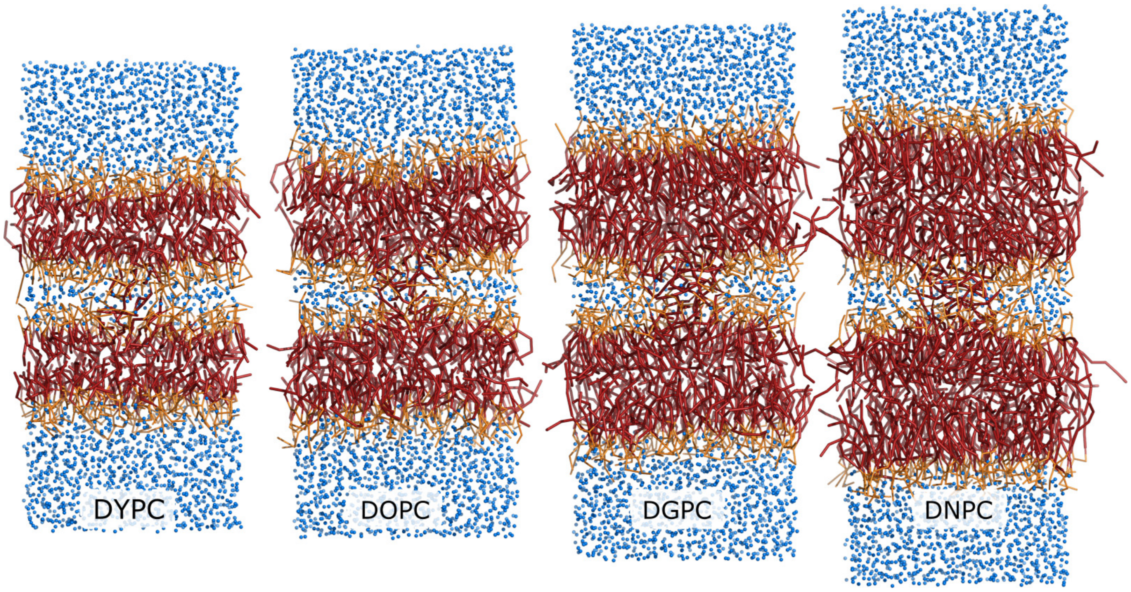

Supplementary Figure 9: Typical simulation frame with an open stalk across membranes with increasing tail length. From left to right: of DYPC, DOPC, DGPC, and DNPC. These MARTINI lipid types correspond approximately to atomistic lipids di-14:1-PC, di-18:1-PC, di-22:1-PC, and di-26:1-PC, respectively. The proximal water compartment contains 6 water beads per nm^2. Frames were taken from the final snapshot of the last umbrella sampling window restrained to $\xi_{ch} = 1$. Lipid tails are shown as dark red sticks, head groups and glycerol region as orange sticks, and water beads as blue spheres. Source data are provided as a Source Data file.

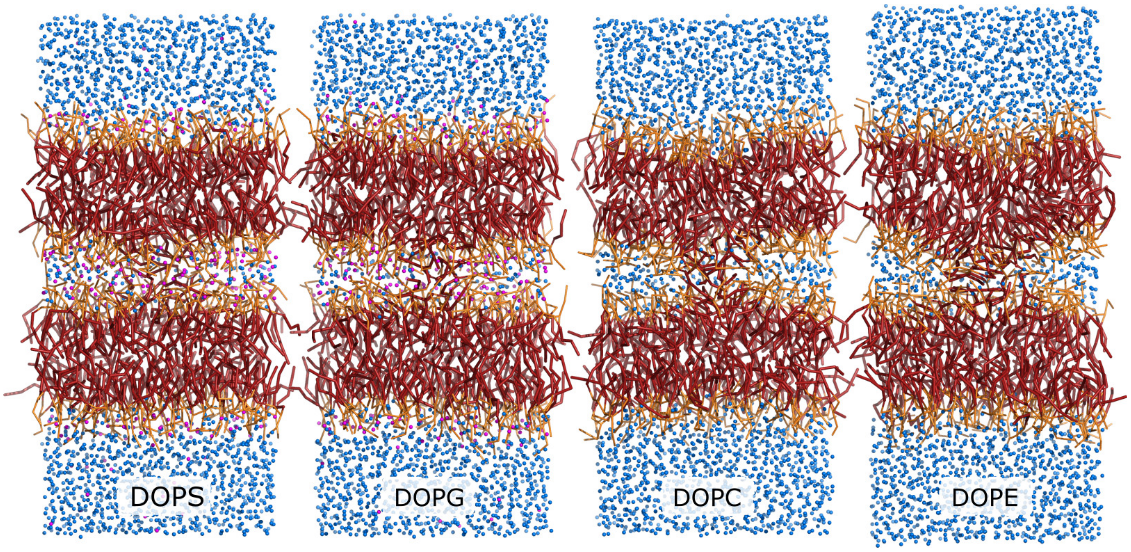

Supplementary Figure 10: Typical simulation frame with an open stalk across membranes of (from left to right) DOPS, DOPG, DOPC, DOPE, taken from the final snapshot of the last umbrella sampling window restrained to $\xi_{ch} = 1$. Lipid tails are shown as dark red sticks, head groups and glycerol beads as orange sticks, water beads as blue spheres, and Na^+ beads as magenta spheres. Source data are provided as a Source Data file.

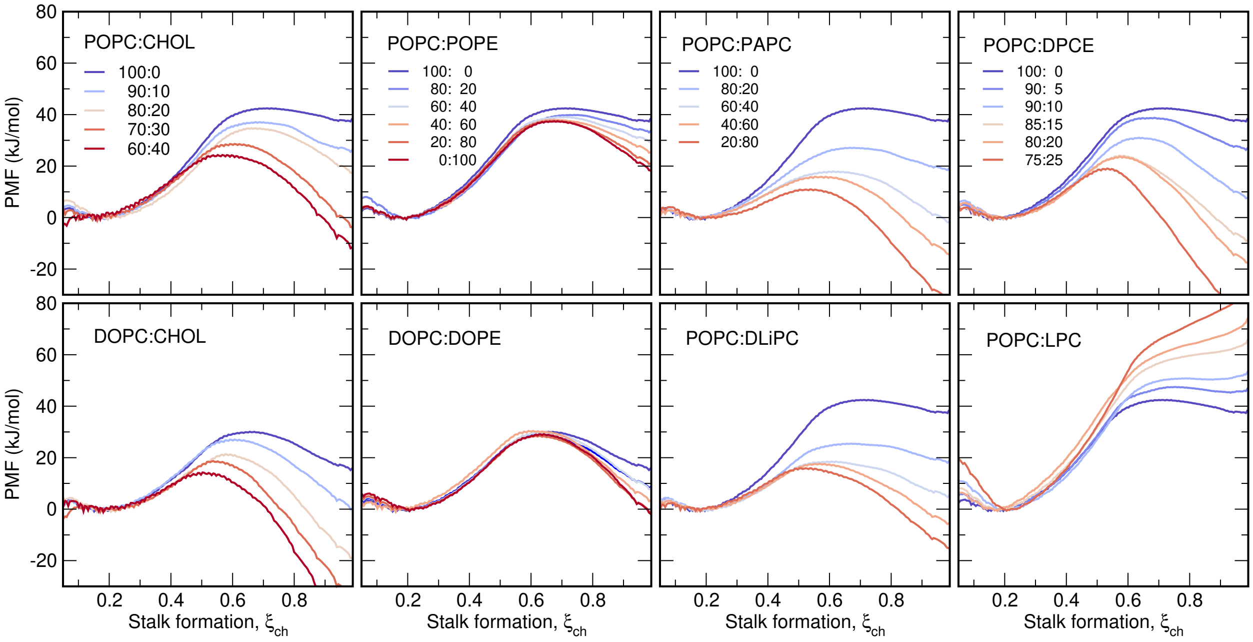

Supplementary Figure 11: Typical PMFs of stalk formation for binary lipid mixtures, as indicated in the figure caption. Lipids are denoted with Martini lipid names. Lipid abbreviations are listed in the legend of Fig. 5. Source data are provided as a Source Data file.

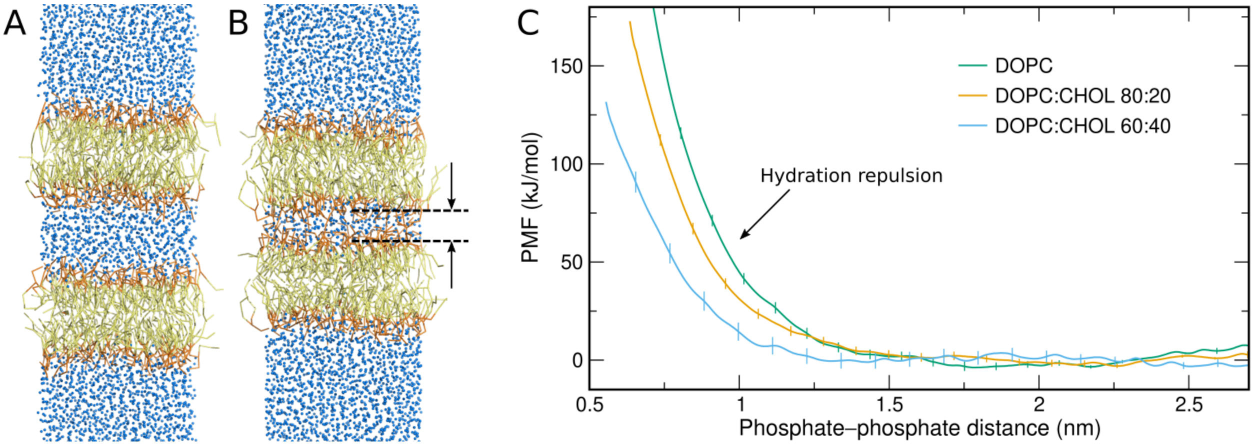

Supplementary Figure 12: PMF calculations of dehydration of the proximal water compartment. (A/B) System with two membranes of pure DOPC at the initial and final distance. (C) PMFs of dehydration with membranes of pure DOPC (green) as well as 80:20 (yellow) or 60:40 (blue) mixtures of DOPC with cholesterol, computed along the center-of-mass distance between the phosphate beads of inner two leaflets. The PMFs confirm that cholesterol reduces the hydration repulsion between the two membranes. Error bars computed by bootstrapping denote 1 SE. Source data are provided as a Source Data file.

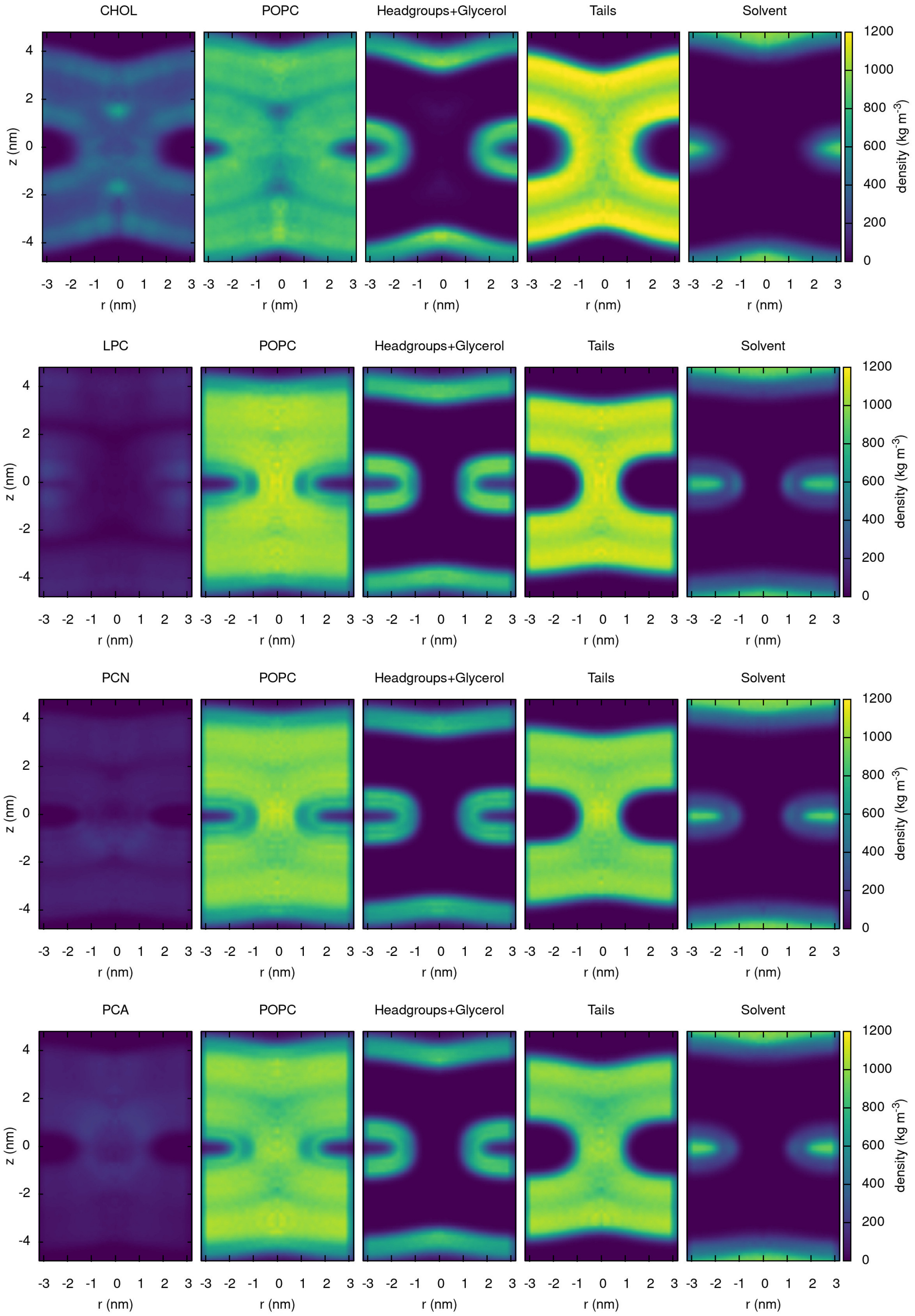

Supplementary Figure 13: Mass densities at lipid stalk (set 1) for systems POPC:Cholesterol 80:20, POPC:LPC 80:20, POPC:PCN 80:20, POPC:PCA 80:20. Densities were computed from the umbrella window restrained to $\xi_{ch} = 1$, omitting the first 50 ns for equilibration. The densities $\rho(r, z)$ were computed as function of the lateral distance r and normal distance z from the center of the stalk, defined as the center of the cylinder used to define ξ_{ch} (see Methods). $\rho(r, z)$ were copied to negative r-values purely for visualization purposes.

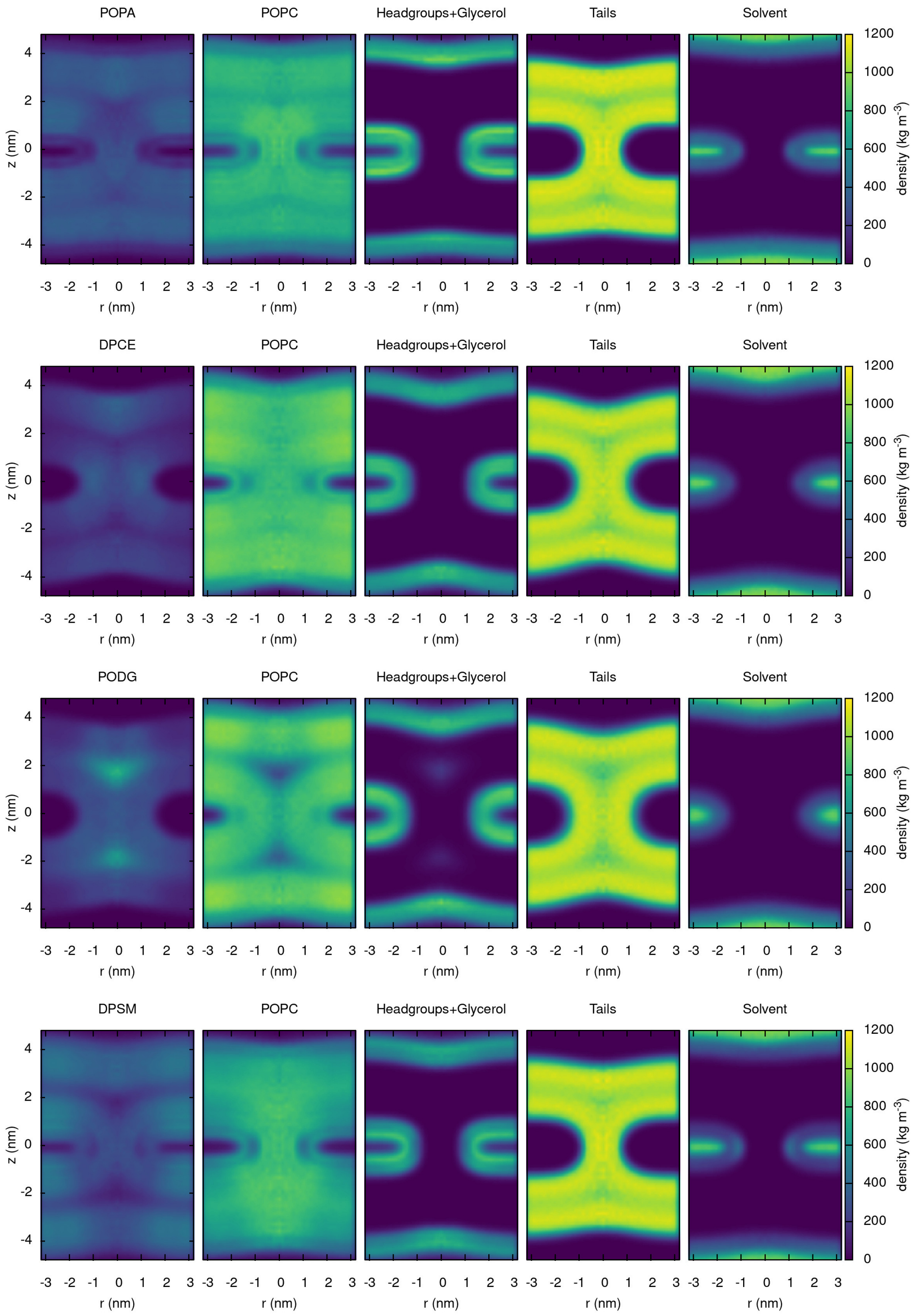

Supplementary Figure 14: Mass densities at lipid stalk (set 2) for systems POPC:POPA 80:20, POPC:DPCE 80:20, POPC:PODG 80:20, POPC:DPSM 60:40.

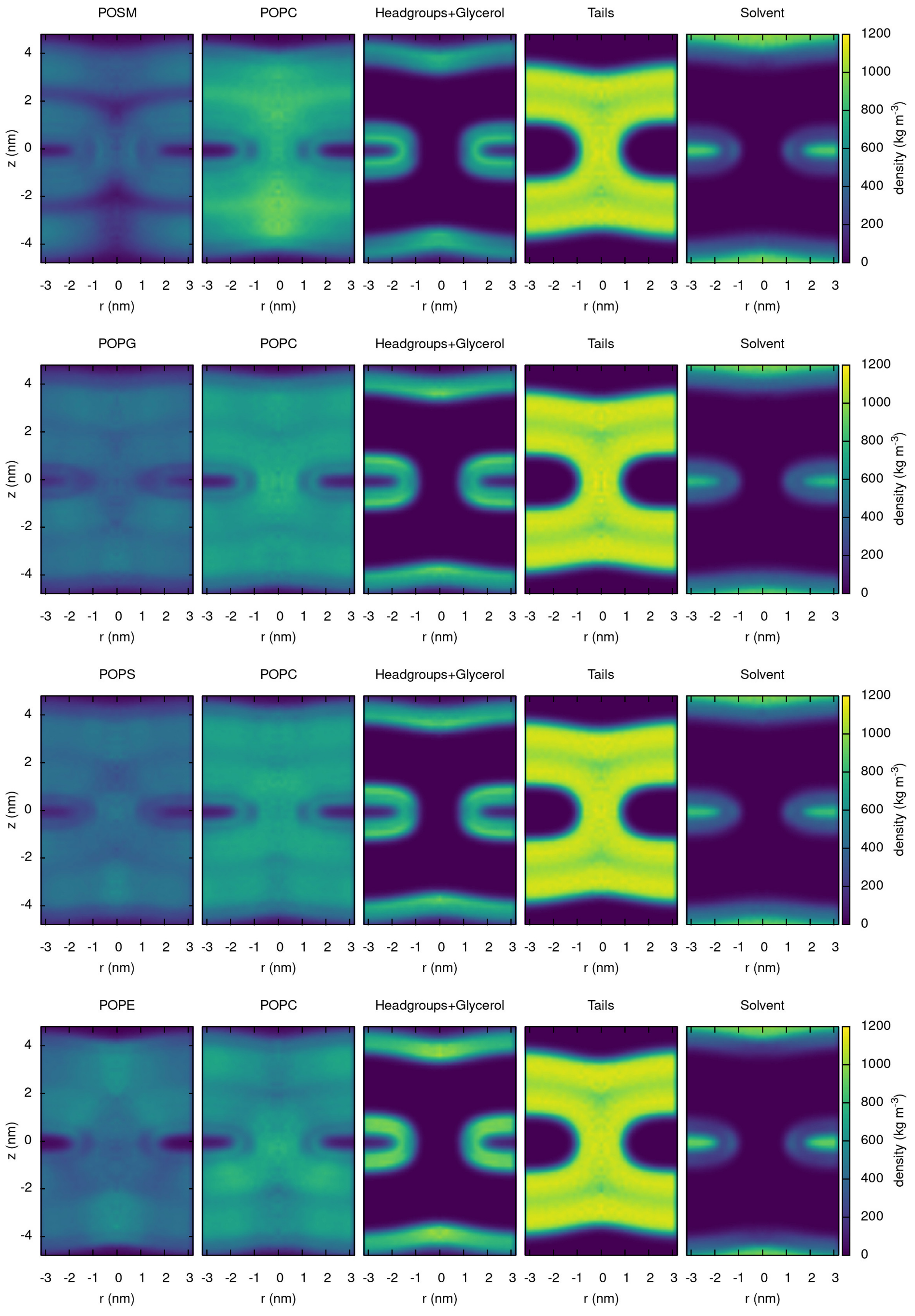

Supplementary Figure 15: Mass densities at lipid stalk (set 3) for systems POPC:POSM 60:40, POPC:POPG 60:40, POPC:POPS 60:40, POPC:POPE 60:40.

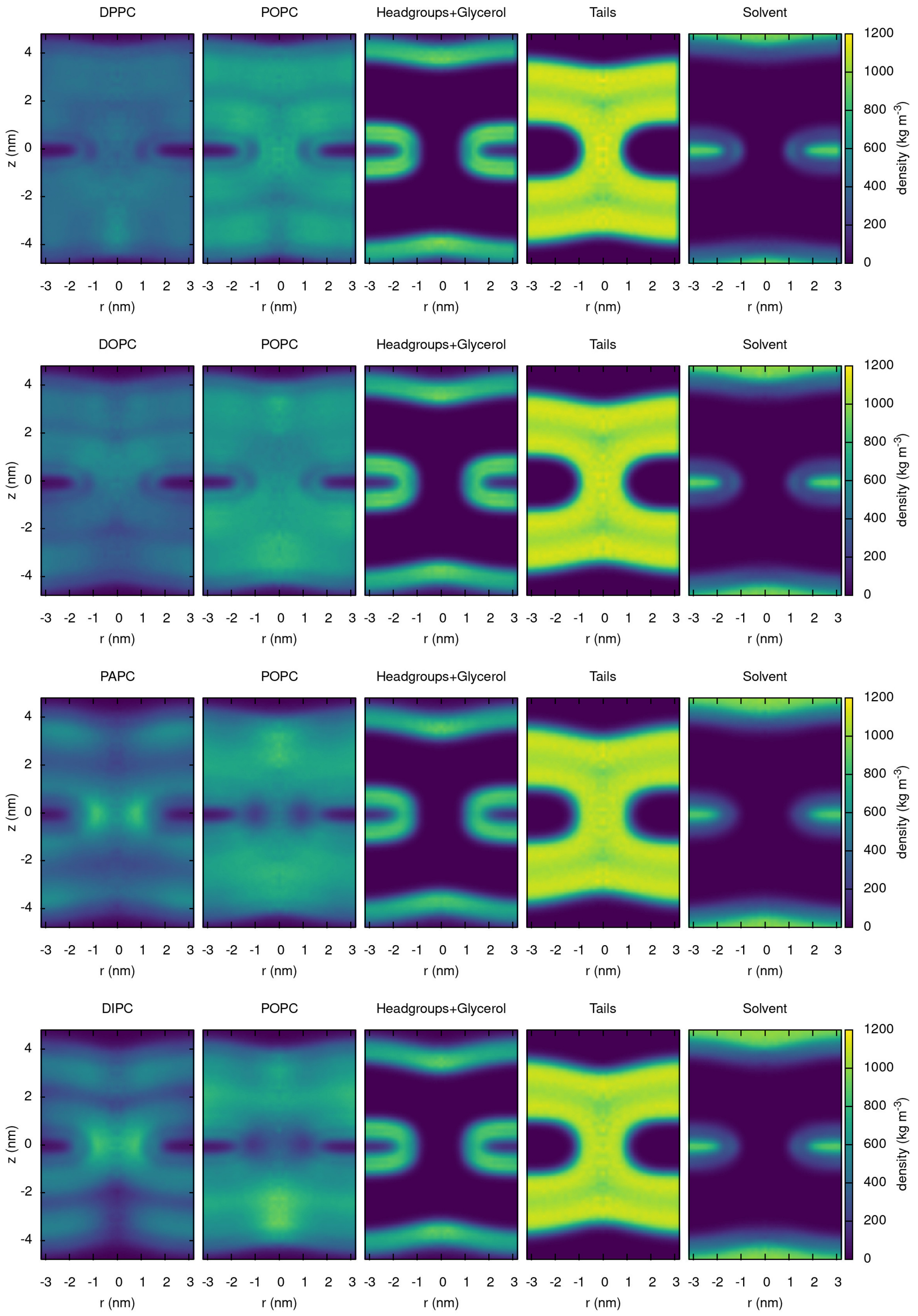

Supplementary Figure 16: Mass densities at lipid stalk (set 4) for systems POPC:DPPC 60:40, POPC:DOPC 60:40, POPC:PAPC 60:40, POPC:DLiPC 60:40 (Martini name: DIPC).

Supplementary Table 1: Lipid composition of plasma membrane models: MARTINI residue name, approximate atomistic correspondence, number of lipids per bilayer for the outer (N_{outer}) and for the inner leaflet (N_{inner}) models. Lipid types are: phosphatidylcholine (PC), sphingomyelin (SM), phosphatidylinositol (PI), phosphatidylethanolamine (PE), cholesterol (CHOL).

MARTINI	∼atomistic	N_{outer}	N_{inner}
DPPC	di-16:0-PC	-	2
POPC	16:0-18:1-PC	6	4
PIPC	16:0-18:2-PC	18	12
PAPC	16:0-20:4-PC	6	-
DPSM	di-16:0-SM	14	2
PNSM	16:0-24:1-SM	8	-
PXSM	16:0-24:0-SM	8	-
PAPI	16:0-20:4-PI	2	2
PAPS	16:0-20:4-PS	4	30
POPE	16:0-18:1-PE	-	2
PAPE	16:0-20:4-PE	2	-
PUPE	16:0-22:6-PE	-	12
OAPE	18:1-20:4-PE	-	4
CHOL		52	52
Total		120	122

Supplementary Table 2: Areas per lipid A_L of the Martini models for DOPC, DOPE, DOPS, and DOPG, computed from umbrella windows with a flat membrane. In Martini, these lipids differ only by the charge as well as by the ε-parameter of the Lennard-Jones potential of the top head group bead type (denoted Q0, Qd, P5, and P4, respectively). DOPC, DOPS, and DOPG adopt similar A_L, implying similar effective size of the headgroup and, hence, a similar geometric shape. DOPE adopt a smaller A_L, implying a smaller effective head groups size, which is compatible with a more cone-shaped geometry. Statistical errors of A_L are approx. 0.03 Å^2.

Martini lipid	A_L (Å^2)
DOPC	68.7
DOPE	65.0
DOPS	68.6
DOPG	67.9

Supplementary Methods

Simulation setup and parameters

The double-membrane systems were set up with a multi-step protocol that ensures the requested degree of hydration between the two membranes, irrespective of the lipid composition. First, a single membrane system was set up with the Insane software.[3] The membrane was hydrated with the requested number of water beads and neutralized with sodium beads, as needed. The energy of the system was minimized, and the membrane was equilibrated for 20 ns to fully relax the box dimensions.

Next, two copies of the membrane were stacked on top of each other, (i) leading to the requested degree of hydration between the proximal leaflets of the double-membrane and (ii) to balanced electric charges between the two water compartments, thereby avoiding a transmembrane electric potential. To fully hydrate the distal leaflets, the box was enlarged along the z direction and additional water was added to the distal water compartment, until the distal leaflets were hydrated with 10 water beads per lipid. The double membrane was equilibrated for another 20 ns. The simulation setup was automated with Bash scripts.

In long simulations of the double-membrane system, we observed occasional membrane permeation by water beads, which would change the degree of hydration of the proximal leaflets. To avoid such permeation, we applied flat-bottomed position restraints (FB-posres) to all water beads. The reference positions of all FB-posres were taken as the center of the box along z. For water in the proximal compartment, an attractive FB-posres was applied, for which the thickness of flat region was set to $z_{\mathrm{fb}} = (z_u - z_l)/2 - 0.5\,\mathrm{nm}$, where z_u and z_l denote the center of mass positions of the upper and lower membrane, respectively. For water in the distal compartment, a repulsive FB-posres was applied, where the repulsive region had a thickness of $(z_u - z_l)/2 + 0.5\,\mathrm{nm}$. The force constant for the quadratic potential was set to $100\,\mathrm{kJ\ mol^{-1}nm^{-2}}$. Together, these FB-posres potentials allowed normal diffusion and fluctuation of water in both compartments and applied only if water beads deeply

penetrated the hydrophobic membrane cores.

Unbiased simulations were carried out with the Gromacs simulation software, version 2020.3.[4] If not stated otherwise, interaction potentials were described with the MARTINI model 2.2.[5] To test the influence of the MARTINI model generation, simulations for certain lipids were also carried out with the beta release 3.2 of Martini 3.0. Neighbor lists were updated with the Verlet scheme. Lennard-Jones and Coulomb potentials were truncated at 1.1 nm. The temperature was controlled at 310 K (if not stated otherwise) through velocity rescaling using four separate coupling groups for the two membranes and for the two water compartments ($\tau = 0.1$ ps).[6] The pressure was kept at 1 bar with the Berendsen barostat ($\tau = 6$ ps).[7] The integration time step was set to 20 fs or 30 fs for simulations with or without cholesterol, respectively.

Visual inspection of the simulation, also after long simulation times, did not reveal any indication of phase separation or lipid demixing (see Fig. 5).

Number of lipids per leaflet and membrane size

To exclude finite-size effects from the periodic boundaries, we computed PMFs of stalk formation for three lipid compositions (pure DOPC, pure DIPC, or a 70:30 mixtures of DOPC with cholesterol) at various membrane sizes (Fig. 1). For the pure-lipid systems, only marginal finite-size effects were evident from the PMFs for very small systems (Fig. 1D, black and orange). Hence, systems without cholesterol were built with 64 lipids per monolayer throughout this study. In contrast, for cholesterol-containing membranes, the stalk free energies were increased by up to 30 kJ/mol for very small box sizes relative to larger boxes, likely because cholesterol renders the membranes more rigid (Fig. 1D, blue). Hence, cholesterol-containing membranes were built with simulation box sizes of approx. 7.5 nm in the x- and y-dimensions (visualized in Fig. 5).

Reaction coordinate for stalk formation

PMFs were computed along the "chain coordinate" ξ_{ch}, which was originally introduced to drive pore formation in membranes.[8,9] ξ_{ch} quantifies the connectivity of two compartments of specific atoms. In this study, ξ_{ch} quantified the degree of connectivity between the hydrophobic cores of two membranes. The shape, radius, lateral position, or lipid composition of the hydrophobic connection were not controlled ξ_{ch} but decided by the force field.

ξ_{ch} was defined by a cylinder of radius 1.2 nm that spans the two hydrophobic regions of the two membranes and the proximal water compartment (Main Article Figure 1A). The cylinder is decomposed into slices with a thickness of 1Å. Then, ξ_{ch} is approximately given by the fraction of slices that are filled by lipid tail beads:

$$\xi_{\text{ch}} = \frac{1}{N_s} \sum_{s=0}^{N_s-1} \delta_\zeta(n_s^{(t)}) \tag{1}$$

Here, N_s is the number of cylinder slices, $n_s^{(t)}$ the number of tail beads in slice s, and δ_ζ is a (differentiable) indicator function that takes $\delta_\zeta = 0$ for an empty slice ($n_s^{(t)} = 0$) and $\delta_\zeta \approx 1$ for a filled slice ($n_s^{(t)} \geq 0$). Hence, upon pulling the system along ξ_{ch}, the slices are filled one-by-one, thereby gradually forming a hydrophobic connection between the two hydrophobic membrane cores, as required for stalk formation. As bead contributing to ξ_{ch}, hydrophobic lipid tail beads as well as hydrophobic beads of cholesterol were used.

Critically, the radius of the cylinder of 1.2 nm does *not* control the radius of the stalk. Instead, the cylinder is merely used to ensure the locality of the hydrophobic protrusions in the membrane plane. If the cylinder radius would be too large, two laterally displaced hydrophobic protrusions, one from the upper and one from the lower membrane, could be misinterpreted as a continuous hydrophobic connection. Such problems would further allow the membrane to evade the energetically unfavorable transition state of stalk formation, which could lead to undesired hysteresis effects.[10] Figure 17 shows that the PMFs depend only marginally on the choice of the cylinder radius in the range between 1.2 nm and 1.6 nm.

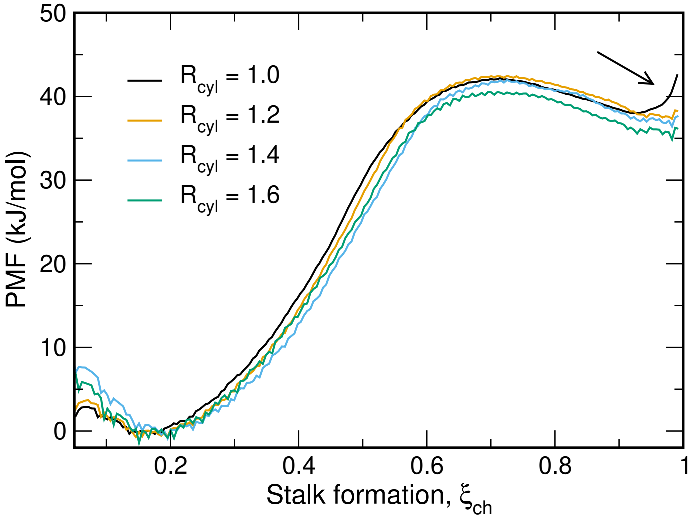

Supplementary Figure 17: PMFs of stalk formation for membranes of pure POPC with 6 water beads per nm^2 in the proximal compartment. PMFs were computed with cylinder radii of 1.0 nm, 1.2 nm, 1.4 nm, or 1.6 nm (see legend). The PMFs depend only marginally on the choice of the cylinder radius. Only for a very small radius of 1.0 nm, the PMF exhibits a spurious increase at $\xi_{\mathrm{ch}} \approx 1$ (black arrow), indicating that hydrophobic beads are "squeezed" into the cylinder. For all larger radii, no such artifact is visible, in line with all other PMFs shown in this study.

The lateral position of the cylinder was not fixed but dynamically defined to allow the cylinder to "follow" the stalk as the stalk travels parallel to the membrane plane. This property excludes that the system moves along ξ_{ch} by shifting the stalk laterally out of the cylinder, which would again lead to undesired hysteresis effects.[11] For implementation details, we refer to previous work.[8] To visualize the stalk in molecular graphics, the stalk was translated to the box center purely for illustration purposes (Figs. 1, 5, 8 9, 10).

To render ξ_{ch} differentiable, the function δ_ζ was defined with a differentiable switch function that approximates and indicator function:[8]

$$\delta_\zeta(x) = \begin{cases} \zeta x & \text{if } x \leq 1 \\ 1 - c\,\mathrm{e}^{-bx} & \text{if } x > 1 \end{cases} \tag{2}$$

Here, the parameter ζ indicates the fraction to which the slice is filled upon the addition of the first apolar bead into the slice. We used $\zeta = 0.75$ in this study. The parameters b and

c are taken as $b = \zeta/(1-\zeta)$ and $c = (1-\zeta)\mathrm{e}^b$, leading to a continuous and differentiable switch function. Likewise, the number of beads $n_s^{(t)}$ in slice s was defined with a differentiable indicator function:

$$n_s^{(t)} = \sum_{j=1}^{N_b} f(\mathbf{r}_j) \quad (3)$$

Here, the sum is taken over all N_b apolar beads and $\mathbf{r}_j$ denotes the Cartesian coordinates of bead j. f is a three-dimensional indicator function that takes unity inside the volume of slice s, and f smoothly switches to zero at the slice boundaries.

The number of slices N_s and thereby the height of the cylinder was chosen depending on the thickness of the proximal water compartment. To this end, the average ξ_{ch} was computed from at last 10 ns of an equilibrium simulation of the flat double membrane using various N_s between 8 and 45 with the 'rerun' functionality of the Gromacs mdrun module. Henceforth, N_s was chosen such that $\xi_{\mathrm{ch}} \approx 0.2$ for the flat membrane. In other words, N_s was chosen such that ~20% of the slices were filled by lipid tail beads in a flat membrane. For membranes of pure POPC and 4 to 18 water beads per nm^2 in the proximal compartment, for instance, this protocol led to cylinders with 16 to 36 slices.

Definition of the differentiable indicator function The indicator function $f(\mathbf{r}_j)$ was formulated as a product of an axial (along z direction) and radial indicator function (in lateral direction), as follows:[8]

$$f(\mathbf{r}_j) = f_{\mathrm{axial}}(z_j; z_s, d)\, f_{\mathrm{radial}}(x_j, y_j; X_{\mathrm{cyl}}, Y_{\mathrm{cyl}}, R_{\mathrm{cyl}}) \quad (4)$$

Here, $\mathbf{r}_j = (x_j, y_j, z_j)$ are the Cartesian coordinates of atom j. X_{cyl} and Y_{cyl} is the position of the cylinder axis in the membrane x-y-plane, and $R_{\mathrm{cyl}} = 1.2$ nm is the radius of the cylinder. z_s is the z coordinate of slice s ($s = 1, \ldots, N_s$), and $d = 0.1$ nm is the thickness of the cylinder slices.

The radial and axial indicator functions are defined with the help the following differen-

tiable step function $\theta(x;h)$, where $2h$ is the width of the switch region:

$$\theta(x;h) = \begin{cases} 1 & \text{if } -1+h \leq x \leq 1-h \\ \frac{1}{2} - \frac{3}{4h}(x-1) + \frac{1}{4h^3}(x-1)^3 & \text{if } 1-h < x < 1+h \\ \frac{1}{2} + \frac{3}{4h}(x+1) - \frac{1}{4h^3}(x+1)^3 & \text{if } -1-h < x < -1+h \\ 0 & \text{otherwise} \end{cases} \tag{5}$$

Hence, $\theta(x;h)$ is zero at $|x| > 1+h$ and unity at $|x| < 1-h$ with continuous switches in the regions $1-h \leq |x| \leq 1+h$. In this work, we used $h = 1/4$. The axial and the radial indicator functions were defined as

$$f_{\text{axial}}(z_j; z_s, d) = \theta\left(\frac{z - z_s}{d/2}; h\right) \tag{6}$$

$$f_{\text{radial}}(x_j, y_j; X_{\text{cyl}}, Y_{\text{cyl}}, R_{\text{cyl}}) = \theta\left(\frac{r_j}{R_{\text{cyl}}}; h\right) \tag{7}$$

where $r_j = [(x_j - X_{\text{cyl}})^2 + (y_j - Y_{\text{cyl}})^2]^{1/2}$ denotes the lateral distance of atom j from the cylinder axis.

The z_s coordinates of the cylinder slices are defined relative to the center of mass Z_{prox} of the proximal water beads, keeping the cylinder centered between the two membranes. The position of the cylinder axis $(X_{\text{cyl}}, Y_{\text{cyl}})$ is defined via a weighted center-of-mass calculation of hydrophobic tail beads in the lateral layer spanned by the cylinder height, i.e., by tail beads whose z coordinate fulfill $Z_{\text{prox}} - N_s d/2 \leq z \leq Z_{\text{prox}} + N_s d/2$. This definition ensures that that cylinder follows the stalk as it travels in the lateral plane. More details as well as the inner derivatives required for computing the forces derived from restraints along ξ_{ch} were described previously.[8]

Umbrella sampling simulations of stalk formation

PMFs were computed using umbrella sampling (US). Initial frames for US were taken from constant-velocity pulling simulations, in which the systems were pulled from $\xi_{\text{ch}} = 0.1$ to $\xi_{\text{ch}} = 1$ within 200 ns, using a force constant of 3000 kJ/mol. Visual inspection of the simulations showed that pulling ξ_{ch} led to gradual stalk formation in all membrane system. 19 umbrella windows were used with reference positions between 0.1 and 1 in steps of 0.05. The force constant was set to 3000 kJ/mol. Each window was simulated for 200 ns, where the first 50 ns were omitted for equilibration. An integration time step of 20 fs was used. All other parameters were chosen as described above. The PMFs were computed with the weighted histogram analysis method (WHAM),[12] as implemented in the gmx wham module of Gromacs.[13]

The free energy of stalk formation ΔG_{stalk} was defined as the value of the PMF at $\xi_{\text{ch}} = 0.95$.

Statistical errors of PMFs and ΔG_{stalk}

Statistical errors were estimated with the Bayesian bootstrap of complete histograms.[13] Accordingly, in each round of bootstrapping, random weights were assigned to all histograms, and the randomly weighted histograms were used to compute a bootstrapped 'synthetic' PMF. Each bootstrapped PMF was defined to zero at $\xi_{\text{ch}} = 0.2$ before computing the standard deviation among the bootstrapped PMFs. The procedure suggested statistical errors in the order of 1 to 3 kJ/mol, indicative of well converged PMFs. Statistical errors of the ΔG_{stalk} values were taken from the statistical error of the PMFs at $\xi_{\text{ch}} = 0.95$.

In addition to the bootstrapping analysis, we computed PMFs from 50 ns time blocks of the umbrella simulations (50–100 ns, 100–150 ns, 150–200 ns). The PMFs agreed within few kilojoules per mole, giving additional support to the convergence of the PMFs. In addition, this analysis suggests that flip-flop events of cholesterol, which occurs on long time scales, could systematically bias the PMFs of cholesterol-containing systems.

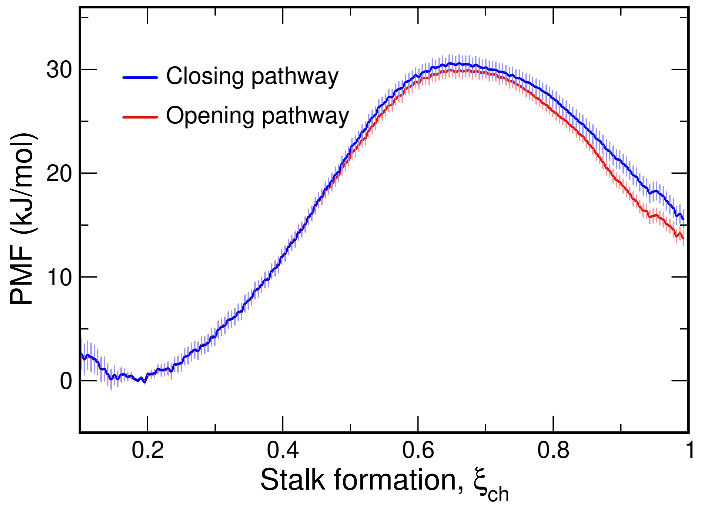

Supplementary Figure 18: PMFs of stalk formation for membranes of pure POPC with 2 water beads per lipid in the proximal compartment. Starting frames for umbrella sampling were taken from constant-velocity pulling simulation conducted either in stalk-opening direction (red) or in stalk-closing direction (blue). The absence of any hysteresis suggests that the PMFs are converged and that the pathways are reversible. Error bars denote 1 SE. Source data are provided as a Source Data file.

Absence of hysteresis between stalk opening and closing pathways

As a test for the validity of the reaction coordinate, we computed the PMFs along stalk-opening and stalk-closing pathways (Fig. 18). We obtained nearly identical PMFs along opening and closing pathways, confirming the absence of undesired hysteresis.

Unbiased simulations of stalk formation and closure

To test whether the PMF along ξ_{ch} reflects the true free energy difference between the flat membrane and the stalk, and to obtain the rates of stalk formation and closure, we carried out unbiased simulations. Here, we used the beta-3.2 release of MARTINI 3.0. We simulated a double-membrane of pure POPC with 230 and 1920 water beads in the proximal and distal water compartments, respectively, for which the PMF suggested a free energy difference between stalk and flat membrane of $\Delta G_{\mathrm{stalk}} \approx 0$ (Main Article Fig. 2). Four replicas of 200 µs each were simulated, which carried out 8 transitions of stalk formation and 7 transitions of stalk closure corresponding to rates of $k_{\mathrm{stalk}} = 16\,\mathrm{ms}^{-1}$ and $k_{\mathrm{closure}} =$

23 ms^{-1}, respectively. Hence, the free simulations suggest a free energy of stalk formation of $\Delta G_{\mathrm{stalk}} = -k_B T \ln(k_{\mathrm{stalk}}/k_{\mathrm{closure}}) = 0.9\,\mathrm{kJ/mol}$, in excellent agreement with the PMF.

Density calculations

The mass density around the stalk was computed with an in-house modification of the Gromacs module gmx density. For the density calculations only, the masses of cholesterol beads were modified to resemble the physical mass distribution, taking the common mapping of three to four heavy atoms onto one CG bead. This step was necessary because the masses of the original MARTINI cholesterol model have been optimized to reproduce the moments of inertia, but not the physical mass distribution. For all other lipids, the original MARTINI mass beads were used.

PMF calculations of dehydration of the proximal leaflet

PMFs for dehydration (Fig. 12) were computed similar to the work by Smirnova *et al.*[1,14] The double-membrane system was setup and equilibrated as described above using 26 water beads per nm^2 and 64 lipid per leaflet. The membranes were composed of pure DOPC or of DOPC/cholesterol mixtures with molar ratios of 80:20 or 60:40. The reaction coordinate ξ_{dehyd} was taken as the center-of-mass distance in z direction between the phosphate beads of the two proximal leaflets (Fig. 12, black arrows). To allow the equilibration of water beads between the two water reservoirs, we introduced a small hole in both leaflets. The hole allowed occasional permeation of water beads, thereby keeping the water beads of the proximal compartment at approximately constant chemical potential.[1,14] We implemented the hole using a repulsive cylindrical flat-bottomed potential acting on all lipid beads with a radius of 0.5 nm and a force constant of $1000\,\mathrm{kJ\,mol^{-1}nm^{-2}}$.

The proximal compartment was dehydrated using constant-velocity pulling along ξ_{dehyd} from ∼3.0 nm to 0.5 nm over 1 µs of simulation with a force constant of $4000\,\mathrm{kJ\,mol^{-1}nm^{-2}}$. The PMFs were computed using umbrella sampling and WHAM[12] with 56 equispaced um-

brella windows at 0.05 nm distance and force constant of 2000 kJ mol^{-1}nm^{-2}. Starting frames were taken from the constant-velocity pulling simulation. Each window was simulated for 3 µs, where the first 1.5 µs were omitted from analysis for equilibration. All simulation parameters were chosen descried above. Statistical errors were computed by bootstrapping complete histograms as described above.[13]

Supplementary Discussion

Attempt frequency of stalk formation coincides with the frequency of headgroup rearrangements along the membrane normal

According to transition state theory, the rate of barrier crossing is given by

$$k = \nu\, \mathrm{e}^{-\Delta G^{\ddagger}/k_B T}, \tag{8}$$

where ν is the attempt frequency and $\Delta G^{\ddagger} = 25\,\mathrm{kJ/mol}$ the barrier height in the PMF (Fig. 2B). With the observed rate of approximately $20\,\mathrm{ms}^{-1}$ and the applied temperature of 310 K, this suggests an attempt frequency of $\nu \approx 0.3\,\mathrm{ns}^{-1}$ or, equivalently, approximately one attempt per $\tau = 1/\nu \approx 3\,\mathrm{ns}$.

As shown in Fig. 19, analysis of the motions of phosphate beads along the membrane normal (z direction) reveals that the lipid headgroups rearrange in normal direction on a similar time scale of ∼3 ns. This agreement of time scales suggests that the attempt frequency for stalk formation obtained with transition state theory may be interpreted as the frequency of head group rearrangements along the membrane normal.

Notably, the time scale for normal displacements of lipids further coincides approximately with the time scale of lateral displacements, which can be defined as the time Δt_{lat} required to diffuse a lipid–lipid distance. From the slope of the mean-square displacement of the phosphate beads during a 50-nanosecond simulation of a POPC membrane, we obtained a

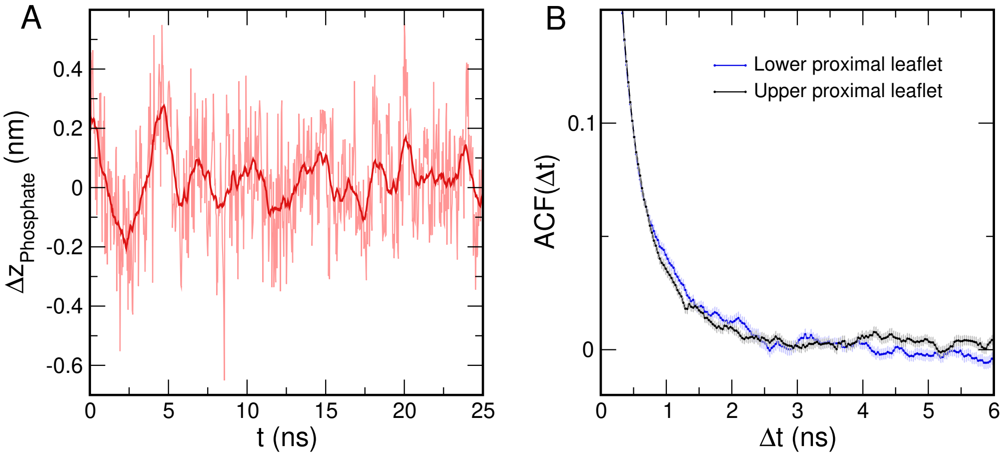

Supplementary Figure 19: Analysis of motions of phosphate beads in the lamellar state along the membrane normal z. (A) Typical z coordinate of a phosphate bead versus simulation time, plotted as Δz relative to the center of mass of all PO4 beads of the same leaflet. The trajectory was taken from the PO4 bead of the first POPC lipid of the simulation system in the first 25 ns of the unbiased simulation of stalk formation (Fig. 2). The dark red line shows the moving average with a window size of 0.93 ns. The smoothed trajectory reveals that, in addition to rapid thermal fluctuations (light red line), the head group carries out frequent conformational rearrangements on the time scale of few nanoseconds, often within 2 to 3 ns (dark red line). (B) Autocorrelation function (ACF) of Δz of all phosphate beads of the lower proximal leaflet (blue) and the upper proximal leaflet (black), computed from a 300ns simulation in the lamellar state. The ACF was first computed for each PO4 bead and subsequently averaged over all beads within the same leaflet. The ACF decays to zero within ∼3 ns, suggesting that the head groups adopt new, statistically independent conformations along the membrane normal within this time scale, in agreement with the visual impression from panel (A). Error bars of the ACFs denote 1 SE, computed from ACFs of the individual lipids ($n = 64$). This analysis demonstrates that the time scale for attempts to form a stalk of ∼3 ns, as obtained from transitions state theory, coincides with the time scale of lipid headgroup rearrangements along the membrane normal. Source data are provided as a Source Data file.

diffusion coefficient of $D = 0.073\,\mathrm{nm}^2/\mathrm{ns}$. We take $\Delta r_{\mathrm{lat}} = A_L^{1/2}$ as a typical lipid–lipid distance, with the area per lipid $A_L = 0.58\,\mathrm{nm}^2$ of the MARTINI POPC model. Using $\langle(\Delta r_{\mathrm{lat}})^2\rangle = 4D\Delta t_{\mathrm{lat}}$, this translates into the time scale of lateral displacements of $\Delta t_{\mathrm{lat}} \approx 2\,\mathrm{ns}$, similar to the time scale obtained for normal displacements (cf. Fig. 19).

Taken together, the conformational sampling of POPC lipids studied here leads to both lateral and normal displacements on similar time scales of few nanoseconds. The lateral displacements manifest in lateral diffusion, whereas the normal displacements may be inter-

preted as attempts for stalk formation with a success rate of $e^{-\Delta G^{\ddagger}/k_B T}$. It is important to note that, because the Martini model leads to a smoothed energy landscape, all dynamics discussed here (for lateral and normal displacements as well as for stalk formation) are likely accelerated relative to atomistic simulations or to experimental conditions.[15]

Supplementary References

(1) Smirnova, Y. G.; Risselada, H. J.; Müller, M. Thermodynamically reversible paths of the first fusion intermediate reveal an important role for membrane anchors of fusion proteins. *Proc Natl Acad Sci USA* **2019**, *116*, 2571–2576.

(2) Endter, L. J.; Smirnova, Y.; Risselada, H. J. Density Field Thermodynamic Integration (DFTI): A "Soft" Approach to Calculate the Free Energy of Surfactant Self-Assemblies. *J. Phys. Chem. B* **2020**, *124*, 6775–6785.

(3) Wassenaar, T. A.; Ingólfsson, H. I.; Böckmann, R. A.; Tieleman, D. P.; Marrink, S. J. Computational lipidomics with insane: a versatile tool for generating custom membranes for molecular simulations. *J. Chem. Theory Comput.* **2015**, *11*, 2144–2155.

(4) Abraham, M. J.; Murtola, T.; Schulz, R.; Páll, S.; Smith, J. C.; Hess, B.; Lindahl, E. GROMACS: High performance molecular simulations through multi-level parallelism from laptops to supercomputers. *SoftwareX* **2015**, *1*, 19–25.

(5) Marrink, S. J.; Risselada, H. J.; Yefimov, S.; Tieleman, D. P.; de Vries, A. H. The MARTINI force field: coarse grained model for biomolecular simulations. *J. Phys. Chem. B* **2007**, *111*, 7812–7824.

(6) Bussi, G.; Donadio, D.; Parrinello, M. Canonical sampling through velocity rescaling. *J. Chem. Phys.* **2007**, *126*, 014101.

(7) Berendsen, H. J. C.; Postma, J. P. M.; DiNola, A.; Haak, J. R. Molecular dynamics with coupling to an external bath. *J. Chem. Phys.* **1984**, *81*, 3684–3690.

(8) Hub, J. S.; Awasthi, N. Probing a continuous polar defect: A reaction coordinate for pore formation in lipid membranes. *J. Chem. Theory Comput.* **2017**, *13*, 2352–2366.

(9) Ting, C. L.; Awasthi, N.; Müller, M.; Hub, J. S. Metastable Prepores in Tension-Free Lipid Bilayers. *Phys. Rev. Lett.* **2018**, *120*.

(10) Hub, J. S. Joint Reaction Coordinate for Computing the Free-Energy Landscape of Pore Nucleation and Pore Expansion in Lipid Membranes. *J. Chem. Theory Comput.* **2021**, *17*, 1229–1239.

(11) Awasthi, N.; Hub, J. S. Simulations of pore formation in lipid membranes: reaction coordinates, convergence, hysteresis, and finite-size effects. *J. Chem. Theory Comput.* **2016**, *12*, 3261–3269.

(12) Kumar, S.; Bouzida, D.; Swendsen, R. H.; Kollman, P. A.; Rosenberg, J. M. The weighted histogram analysis method for free-energy calculations on biomolecules. I. The method. *J. Comput. Chem.* **1992**, *13*, 1011–1021.

(13) Hub, J. S.; Groot, B. L. d.; Spoel, D. v. d. g_wham–A Free Weighted Histogram Analysis Implementation Including Robust Error and Autocorrelation Estimates. *J. Chem. Theory Comput.* **2010**, *6*, 3713–3720.

(14) Smirnova, Y. G.; Aeffner, S.; Risselada, H. J.; Salditt, T.; Marrink, S. J.; Müller, M.; Knecht, V. Interbilayer repulsion forces between tension-free lipid bilayers from simulation. *Soft Matter* **2013**, *9*, 10705.

(15) Marrink, S. J.; Tieleman, D. P. Perspective on the Martini model. *Chem. Soc. Rev.* **2013**, *42*, 6801.
