# Supplementary material for: Free energies of membrane stalk formation from a lipidomics perspective
Source: Nat Commun. 2021 Nov 15;12:6594. doi: 10.1038/s41467-021-26924-2 (PMC8593120; doi:10.1038/s41467-021-26924-2)
Supplement: Supplementary file 3 — Reporting Summary [file 41467_2021_26924_MOESM3_ESM.pdf]

nature portfolio

Corresponding author(s): Jochen S. Hub

Last updated by author(s): Sept 27, 2021

Reporting Summary

Nature Portfolio wishes to improve the reproducibility of the work that we publish. This form provides structure for consistency and transparency in reporting. For further information on Nature Portfolio policies, see our Editorial Policies and the Editorial Policy Checklist.

Statistics

For all statistical analyses, confirm that the following items are present in the figure legend, table legend, main text, or Methods section.

n/a	Confirmed	
☐	☒	The exact sample size (*n*) for each experimental group/condition, given as a discrete number and unit of measurement
☒	☐	A statement on whether measurements were taken from distinct samples or whether the same sample was measured repeatedly
☒	☐	The statistical test(s) used AND whether they are one- or two-sided<br>*Only common tests should be described solely by name; describe more complex techniques in the Methods section.*
☒	☐	A description of all covariates tested
☒	☐	A description of any assumptions or corrections, such as tests of normality and adjustment for multiple comparisons
☐	☒	A full description of the statistical parameters including central tendency (e.g. means) or other basic estimates (e.g. regression coefficient) AND variation (e.g. standard deviation) or associated estimates of uncertainty (e.g. confidence intervals)
☒	☐	For null hypothesis testing, the test statistic (e.g. *F*, *t*, *r*) with confidence intervals, effect sizes, degrees of freedom and *P* value noted<br>*Give P values as exact values whenever suitable.*
☐	☒	For Bayesian analysis, information on the choice of priors and Markov chain Monte Carlo settings
☒	☐	For hierarchical and complex designs, identification of the appropriate level for tests and full reporting of outcomes
☒	☐	Estimates of effect sizes (e.g. Cohen's *d*, Pearson's *r*), indicating how they were calculated

Our web collection on statistics for biologists contains articles on many of the points above.

Software and code

Policy information about availability of computer code

Data collection	GROMACS, versions 2020.3 and 2018.8. In-house modification of GROMACS at https://gitlab.com/cbjh/gromacs-chain-coordinate
Data analysis	bash 5.0.17 and 5.1.4, GROMACS 2020.3 and 2018.8, gnuplot 5.4, xmgrace 5.1.25_5

For manuscripts utilizing custom algorithms or software that are central to the research but not yet described in published literature, software must be made available to editors and reviewers. We strongly encourage code deposition in a community repository (e.g. GitHub). See the Nature Portfolio guidelines for submitting code & software for further information.

Data

Policy information about availability of data

All manuscripts must include a data availability statement. This statement should provide the following information, where applicable:

- Accession codes, unique identifiers, or web links for publicly available datasets
- A description of any restrictions on data availability
- For clinical datasets or third party data, please ensure that the statement adheres to our policy

Source data used to render the figures are provided with this paper. Simulation systems (starting conformations, topologies, MD parameter files) are available open access at zenodo.org (doi: 10.5281/zenodo.5196036) via https://doi.org/10.5281/zenodo.5196036. Additional data that support the findings of this study are available from the corresponding author upon reasonable request.

Field-specific reporting

Please select the one below that is the best fit for your research. If you are not sure, read the appropriate sections before making your selection.

☒ Life sciences ☐ Behavioural & social sciences ☐ Ecological, evolutionary & environmental sciences

For a reference copy of the document with all sections, see nature.com/documents/nr-reporting-summary-flat.pdf

Life sciences study design

All studies must disclose on these points even when the disclosure is negative.

Sample size	Simulation system sizes was chosen large enough to exclude periodic boundary artifacts. The simulation time (and thereby the number of independent simulation frame samples) was chosen large enough (200ns per umbrella window) to ensure that error bars were on the order of only few kilojoule per mole.
Data exclusions	No data was excluded. In rare cases, the double-membranes were unstable during equilibration, as evident from visual inspection, because they start from an energetically unfavorable ordered conformation. In such cases the equilibration was repeated.
Replication	All attempts of replicating simulation results were successful. Many PMFs were computed from independent time blocks of 50ns, providing three independent samples for each PMFs. The PMFs computed from 50-nanosecond time blocks agreed in all cases within few kilojoule per mole.
Randomization	Randomization do not change the outcome of our simulations.
Blinding	Blinding does not change the outcome of our simulations.

Reporting for specific materials, systems and methods

We require information from authors about some types of materials, experimental systems and methods used in many studies. Here, indicate whether each material, system or method listed is relevant to your study. If you are not sure if a list item applies to your research, read the appropriate section before selecting a response.

Materials & experimental systems

n/a	Involved in the study
☒	☐ Antibodies
☒	☐ Eukaryotic cell lines
☒	☐ Palaeontology and archaeology
☒	☐ Animals and other organisms
☒	☐ Human research participants
☒	☐ Clinical data
☒	☐ Dual use research of concern

Methods

n/a	Involved in the study
☒	☐ ChIP-seq
☒	☐ Flow cytometry
☒	☐ MRI-based neuroimaging
