# Supplementary figures and images for: Free energies of membrane stalk formation from a lipidomics perspective
Source: Nat Commun. 2021 Nov 15;12:6594. doi: 10.1038/s41467-021-26924-2 (PMC8593120; doi:10.1038/s41467-021-26924-2)

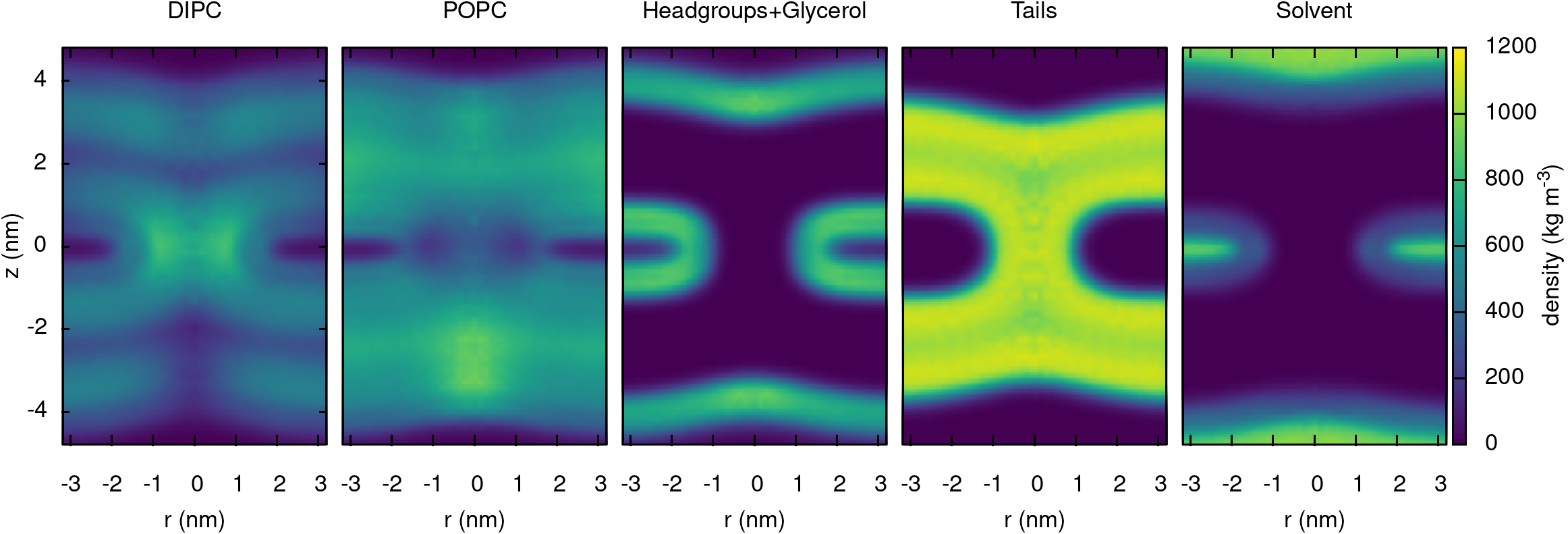

Supplement: Supplementary file 4 — Source Data [file 41467_2021_26924_MOESM4_ESM.zip › SI_data/Fig6/all-densities.DIPC.png]

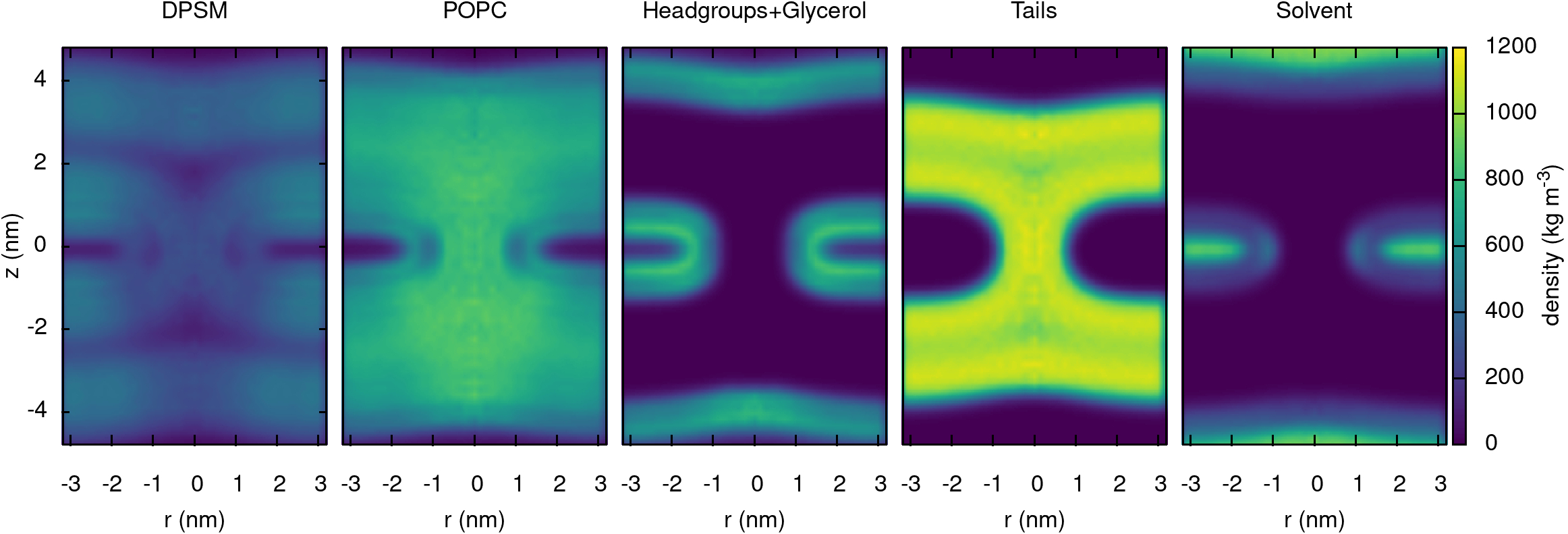

Supplement: Supplementary file 4 — Source Data [file 41467_2021_26924_MOESM4_ESM.zip › SI_data/Fig6/all-densities.DPSM.png]
